# Supplementary material for: Analysis of PPARγ Signaling Activity in Psoriasis
Source: Int J Mol Sci. 2021 Aug 10;22(16):8603. doi: 10.3390/ijms22168603 (PMC8395241; doi:10.3390/ijms22168603)
Supplement: Supplementary file 1 [file ijms-22-08603-s001.zip › Supplemental materials_Analysis of PPARg signaling activity in psoriasis/Pathway models/Models images and html files/PPARG inhibits phenotypes assotiated with psoriasis/134205.html]

inflammatory infiltrate


# Disease inflammatory infiltrate

|  |  |
| --- | --- |
| URN | urn:agi-disease:inflammatory%20infiltrate |
| Total Entities | 0 |
| Connectivity | 1578 |
| Name | inflammatory infiltrate |

---

|  |  |
| --- | --- |
| Pathway | PPARG inhibits phenotypes assotiated with psoriasis |
|  | efects selected |

---

|  |  |
| --- | --- |
| MedScan ID | 9603857 |

---

|  |  |
| --- | --- |
| Alias | inflammation infiltrate |
|  | inflammatory infiltrates |
|  | inflammation infiltrates |
|  | inflammatory infiltrate |
|  | inflammation infiltrating |
|  | inflammatory infiltrating |

---
